# Around the Hospitals

**Published:** 1893-09-30

**Authors:** 


### Around the Hospitals.

Notice to the Managers of Institutions.—Special space is now reserved for the insertion of notices of hospital meetings and festivals. The Editor requests that all notices may reach The Hospital Office, 428, Strand, at least one week before the date of each meeting.

**Salisbury Infirmary.**—This institution has done good work during the year past. The annual report shows an increase in the number of both in and out-patients, 889 of the former having been admitted to the wards, as against 826 in the preceding year, whilst 1,690 of the latter have been treated, as compared with 1,506 in 1892. The hospital has lost the services of the late able matron, Miss Carvosso, who resigned in order to take a similar appointment at the Derbyshire Royal Infirmary. The committee expressed to Miss Carvosso on her departure, on behalf of the governors, their appreciation of her services during her three years of office, and their regret at her resignation. The post thus left vacant has been filled by the appointment in February last of Miss Amy Johnstone. We are sorry to see that a recommendation of the committee to increase the matron's salary to £90 per annum was not adopted by the annual Court last year. We also regret to find that the authorities are in some financial difficulties, and funds are not forthcoming to clear off the debts incurred through recent additions and improvements to the building. The alterations were badly needed, and it is to be hoped the people of Salisbury and the neighbourhood will not allow this state of things to last, more especially as further improvements are urgent in the nurses' quarters, and should be set about forthwith. The committee have granted to the head nurse, who has faithfully served the hospital for 27 years, the largest allowance in their power to confer, 10s. per week, "in testimony of their high regard." Votes of thanks are recorded to the medical and surgical staff and the resident and nursing staff of the infirmary. Thirty-three patients have been sent to the Herbert Convalescent Home at Bournemouth during the year. It is proposed to hold a bazaar in aid of the infirmary in November, and we cordially hope this may result in a successful addition to the funds.

**St. Bartholomew's Hospital.**—This hospital, occupying the unique position of opulence which it does, and being without need to supply the public with details of work done, presents a very uninteresting report of its management. How or at what cost its great and beneficent work is done we are not told, but the figures which are given show that no less than 5,953 in-patients and 158,888 out-patients were treated within its walls. No report of progress or alterations are given since 1882, and yet in the nursing department alone important alterations have been made in the accommodation since the year 1887. At that time some of the sleeping accommodation and sanitary arrangements for the nurses were a disgrace to any institution. One room, originally part of a shop, with the large frontage of windows partly boarded up, was so damp that the clothes of the nurse, kept in her box in winter near the fire, were actually mildewed, and damp was only too visible to the eye on many walls. Since then great improvements have been made in the sleeping accommodation, and the nurses' sitting-room, then little better than a third-class waiting-room at a railway station, is now a comfortable and adequate apartment. Other improvements throughout the hospital have taken place, which it would be interesting to the public and just to the hospital to publish in its report.

